# In Vitro Liver Toxicity Testing of Chemicals: A Pragmatic Approach

**DOI:** 10.3390/ijms22095038

**Published:** 2021-05-10

**Authors:** Andrés Tabernilla, Bruna dos Santos Rodrigues, Alanah Pieters, Anne Caufriez, Kaat Leroy, Raf Van Campenhout, Axelle Cooreman, Ana Rita Gomes, Emma Arnesdotter, Eva Gijbels, Mathieu Vinken

**Affiliations:** Department of Pharmaceutical and Pharmacological Sciences, Vrije Universiteit Brussel, Laarbeeklaan 103, 1090 Brussels, Belgium; andres.tabernilla.garcia@vub.be (A.T.); bruna.dos.santos.rodrigues@vub.be (B.d.S.R.); alanah.pieters@vub.be (A.P.); anne.caufriez@vub.be (A.C.); kaat.leroy@vub.be (K.L.); raf.van.campenhout@vub.be (R.V.C.); axelle.cooreman@vub.be (A.C.); ana.rita.coelho.gomes@vub.be (A.R.G.); emma.arnesdotter@vub.be (E.A.); eva.anne.gijbels@vub.be (E.G.)

**Keywords:** liver, in vitro, cytotoxicity, liver-specific toxicity, mechanisms

## Abstract

The liver is among the most frequently targeted organs by noxious chemicals of diverse nature. Liver toxicity testing using laboratory animals not only raises serious ethical questions, but is also rather poorly predictive of human safety towards chemicals. Increasing attention is, therefore, being paid to the development of non-animal and human-based testing schemes, which rely to a great extent on in vitro methodology. The present paper proposes a rationalized tiered in vitro testing strategy to detect liver toxicity triggered by chemicals, in which the first tier is focused on assessing general cytotoxicity, while the second tier is aimed at identifying liver-specific toxicity as such. A state-of-the-art overview is provided of the most commonly used in vitro assays that can be used in both tiers. Advantages and disadvantages of each assay as well as overall practical considerations are discussed.

## 1. Introduction

The liver is a primary target for systemic toxicity caused by chemicals, which results from its particular function and location in the organism. Chemical-induced liver toxicity usually arises from combined general cell type-nonspecific cytotoxic and liver tissue-specific toxic actions. Throughout the research field of liver toxicity, most attention has yet been paid to pharmaceutical chemicals. In fact, drug-related liver toxicity accounts for more than 50% of all clinical cases of acute liver failure [1], being responsible for 6% of all liver-related deaths and for 7% of all liver transplantations [2]. Furthermore, drug-induced liver injury is a major reason of drug failure during pre-marketing and post-marketing phases of drug development, accounting for up to 29% of all drug withdrawals [3]. Especially in the past 2 decades, it has become clear that chemicals from other sectors equally have the potential to cause liver toxicity, including, but not limited to, industrial chemicals, biocides, cosmetic ingredients, food additives and dietary supplements [4,5]. This not only raises human health issues, but may also have financial repercussions for the industries involved. For these reasons, it is of utmost importance to identify liver toxic potential of chemicals early on in order to secure safe exposure to humans. Historically, animal testing has been used as the basis for such safety evaluation exercises. This allows to identify the most relevant and sensitive adverse effect, which is used to characterise the so-called point-of-departure in the dose–response curve for setting safety limits for humans [6]. Although uncertainty is considered in extrapolation of results between species, the assumption is made that the adverse effect described in the laboratory animal will equally occur in human [7].

Nevertheless, effects not seen in laboratory animals frequently appear in humans and vice versa, which underscores the relevance of interspecies differences. Driven by such scientific constraints as well as the obvious ethical reasons, there is an increasing tendency to address human-based animal-free methods for safety evaluation of chemicals. This paradigm shift is a cornerstone of the seminal report entitled “Toxicity testing in the 21st century: a vision and a strategy” issued by the US National Academy of Sciences in 2007. This document advocates reduced reliance on apical toxicological outcome testing in laboratory animals and strongly encourages the use of human-based non-animal methods, such as in vitro experimentation, designed to detect perturbations in toxicity pathways [8]. The present manuscript describes a pragmatic strategy that fully aligns with this concept, by proposing a tiered approach for the in vitro testing of liver toxicity. The scope of this strategy is broad, as the underlying rationale and modus operandi can be de facto applied to any kind of chemical for which liver toxicity testing is warranted. In the first part, a short recapitulation of liver structure and function is provided. In the second and third part, principles and mechanisms of general cytotoxicity as well as the most commonly used in vitro assays to study general cytotoxicity are discussed (Table 1). The fourth and fifth parts revise liver-specific toxicity and liver-specific toxicity methods, respectively (Table 2). The sixth part discusses a number of practical aspects that should be taken into consideration when setting up in vitro liver toxicity testing schemes.

## 2. Liver Structure and Function

**Figure 1:**
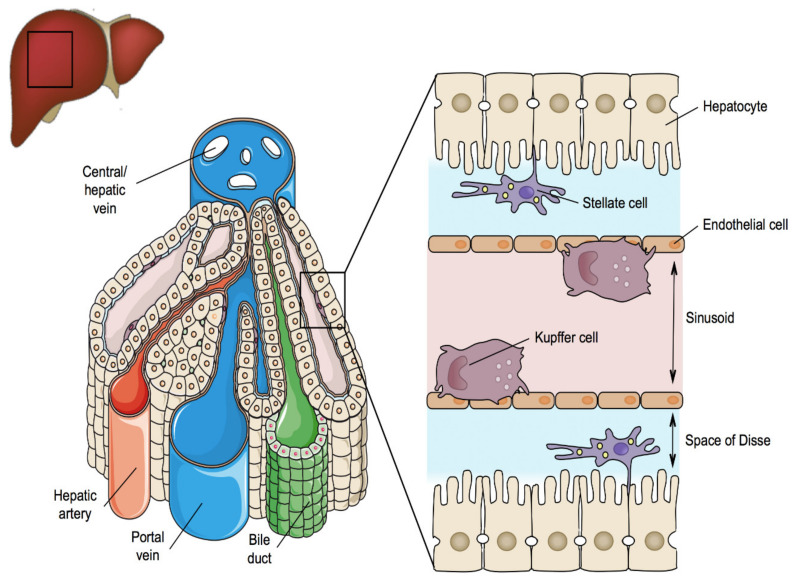
Scheme representing the structure of the liver, highlighting the cellular architecture of its functional unit, the acinus.

The liver is located in the upper right quadrant of the abdominal cavity and consists of 4 lobes. The liver has a unique 2-way blood supply. It receives blood rich in digested nutrients from the entire gastrointestinal tract as well as from the spleen and pancreas via the portal vein. The liver also receives oxygen-rich blood from the aorta through the hepatic artery [176,177,178]. Within the liver, blood vessels subdivide into small capillaries, called sinusoids, leading to a lobule that drains towards the central hepatic vein. The lobule is the morphological unit of the liver and has a hexagonal structure that is composed of plates of hepatocytes separated by sinusoids [179,180]. The functional unit of the liver is the acinus and delineates the elliptical region of hepatocytes from adjacent lobules (Figure 1). As such, 3 acinar zones can be distinguished corresponding with the distance from the arterial blood supply, namely the periportal, the midlobular and the perivenous zones [181]. Depending on the location in these areas, hepatocytes have different oxygen and nutrient supplies. As a consequence, differential expression of liver-specific genes occurs throughout the acinus, which underlies local variation in functionality, a phenomenon known as zonation [182].

The true workhorses of the liver are the parenchymal cells or hepatocytes [180]. They take care of most of the liver-specific functions, such as metabolism of carbohydrates and lipids, bile acid (BA) production, production of albumin and blood coagulation factors, and biotransformation of xenobiotics. Hepatocytes have a polygonal shape, about 25 µm in diameter, and constitute as much as 60% of the total amount of cells in the liver. Hepatocytes are highly polarized epithelial cells with 3 different plasma membrane domains, namely the sinusoidal, the lateral and the canalicular poles. The sinusoidal area is in contact with liver sinusoidal endothelial cells, while the canalicular zone aligns with bile canaliculi. The lateral area is the zone between 2 neighbouring hepatocytes [183].

Liver sinusoidal endothelial cells represent up to 20% of all liver cells. They are a particular type of endothelial cells because they lack a basal lamina, possess fenestrae and can transfer molecules and particles by endocytosis [184]. These cells form a continuous line of liver sinusoids and by doing so, they create a space, called the space of Disse, between the hepatocytes and the blood [185,186].

Hepatic stellate cells (HSCs) make up about 5% of the liver cell population. They generate extracellular matrix (ECM) components, control microvascular tone, and store vitamin A and triglycerides. In healthy liver, HSCs are in a quiescent state. Upon liver damage, however, they transform into myofibroblast-like cells, which accumulate excessive amounts of ECM constituents [187]. This so-called scarring process lies at the basis of liver fibrosis [188].

Kupffer cells represent around 15% of the total liver cell number and are located within the sinusoidal lumen. Kupffer cells are irregular in shape and possess an important phagocytic function, which is indispensable for the clearance of large particles, such as bacteria, damaged hepatocytes and erythrocytes. Kupffer cells also produce chemokines and cytokines [189,190,191].

## 3. Mechanisms of General Cytotoxicity

**Figure 2:**
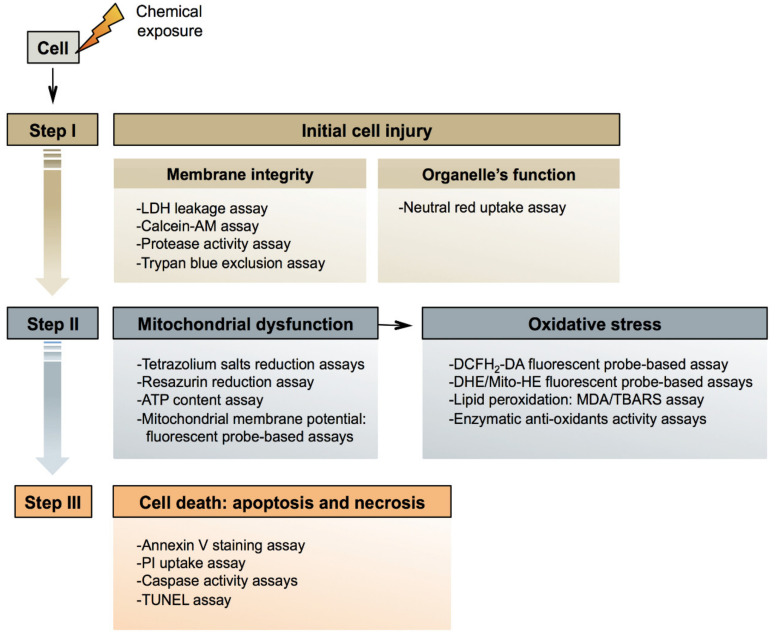
Scheme representing the mechanisms of general cytotoxicity and corresponding in vitro assays. ATP: adenosine triphosphate; Calcein-AM: calcein-acetoxymethyl; DCFH_2_-DA: 2′,7′-dichlorodihydrofluorescein diacetate; DHE: dihydroethidium; LDH: lactate dehydrogenase; MDA: malondialdehyde; PI: propidium iodide; TBARS: thiobarbituric acid reactive substance; TUNEL: terminal deoxynUcleotidyl transferase dUTP Nick End Labeling.

Cytotoxicity denotes the ability of a chemical to damage living cells, in particular by compromising functional and structural features related to general cellular housekeeping. Being a comprehensive process that can occur in any type of cell, the pathways underlying cytotoxicity are generic [192,193,194]. As such, 3 consecutive steps can be distinguished in cytotoxicity (Figure 2). The first step involves initial cell injury. In the second step, mitochondrial dysfunction takes place, leading to cell death in the third step [195].

### 3.1. Initial Injury

A first common mechanism of initial injury is destruction of the cell plasma membrane. The double phospholipid layer in the plasma membrane provides physical segregation between the extracellular environment and the cytosol, which contributes to selective passage of substances between both compartments. Damage to the plasma membrane triggered by chemicals can occur in a number of ways, such as by the accumulation and binding to the phospholipid bilayer, called narcosis [196].

A second mechanism of initial injury relates to interfering with subcellular architecture. Cellular functions are restricted to specific organelles within the cell, such as the rough endoplasmic reticulum, taking care of protein synthesis, and the nucleus, where genetic material is stored. This strict compartmentalization can be affected by chemicals, which in turn jeopardises cellular functionality [193,194].

### 3.2. Mitochondrial Dysfunction

Mitochondria form the energy core of each cell. The process of generating cellular energy is complex and driven by a network of entangled pathways. Pyruvate is taken up by mitochondria and transformed into acetylcoenzyme A. Simultaneously, fatty acids (FAs) bound to acetylcoenzyme A enter the mitochondria, and are split by successive β-oxidation cycles, also yielding acetylcoenzyme A. The latter is then converted into carbon dioxide through the tricarboxylic acid cycle, which produces nicotinamide adenine dinucleotide (NADH) and flavin adenine dinucleotide. Both these molecules become oxidised, thus generating electrons that are used to reduce molecular oxygen to water. This reaction, catalysed by respiratory chain complexes, is associated with the extrusion of protons from the matrix into the inner membrane space of mitochondria. When energy is needed, protons re-enter the matrix to generate adenosine triphosphate (ATP) from adenosine diphosphate [197].

Chemicals can compromise mitochondrial functionality due to alterations in the mitochondrial membrane potential, uncoupling of the mitochondrial respiratory chain leading to production of oxidative stress species and cellular oxidative stress damage, and inhibition of ATP synthesis [198,199,200]. Additionally, chemicals can affect the mitochondrial permeability transition pore, which is a complex megachannel that spans across the inner and outer membranes of mitochondria [201,202]. Opening of the mitochondrial permeability transition pore leads to the release of cytochrome C into the cytosol or the uptake of protons and water in the mitochondrial matrix. In case the mitochondrial permeability transition pore opens abruptly in a large number of mitochondria, drastic ATP depletion takes place. This is deleterious for several cellular functions that rely on energy and results in the disequilibrium of ion levels, eventually causing necrotic cell death. In case the mitochondrial permeability transition pore opens in a small number of mitochondria, unaffected mitochondria continue to generate ATP, whereas disrupted mitochondria release cytochrome C. This triggers apoptotic cell death [201,203,204].

### 3.3. Cell Death

Programmed cell death, also called apoptosis, is driven by 2 major pathways, namely the extrinsic pathway and the intrinsic pathway. The intrinsic pathway starts by stimulation of the release of cytochrome C from mitochondria, which is controlled by pro-apoptotic and anti-apoptotic B-cell lymphoma 2 proteins. Cytochrome C forms an apoptosome with deoxyadenosine triphosphate, apoptotic protease activating factor 1 and procaspase 9, which activates caspase 3. The extrinsic pathway is prompted by the binding of specific ligands, such as Fas ligand, to their receptors at the cell plasma membrane surface. This promotes the cleavage of procaspase 8, which subsequently triggers caspase 3. The outcome of both the intrinsic and extrinsic pathways thus is the activation of caspase 3, which is the main executor of apoptosis. Caspase 3 indeed cleaves a broad variety of cellular proteins, including cytoskeletal proteins, giving rise to the typical apoptotic phenotype, involving cell shrinkage, blebbing, cytoplasmic and nuclear condensation, deoxyribonucleic acid (DNA) fragmentation and the formation of apoptotic bodies [205,206,207,208,209,210,211].

Necrosis, unlike apoptosis, is a rather chaotic and passive process caused by a wide spectrum of stress factors. Necrosis typically starts with the loss of ion homeostasis, which activates several enzyme systems, including proteases, phospholipases and endonucleases. This results in cell swelling, cell lysis and induction of inflammation [206,207,209,211,212,213].

## 4. General Cytotoxicity In Vitro Methods

### 4.1. Membrane Integrity Assays

#### 4.1.1. LDH Leakage Assay

The lactate dehydrogenase (LDH) leakage assay is an indirect method used to detect the loss of cell membrane integrity by means of assessing the LDH extracellular activity upon damage in the cell plasma membrane [214,215]. LDH is an enzyme present in all cell types, which catalyses the interconversion of pyruvate and lactate with concomitant interconversion of NAD and NADH. This process can be indirectly monitored by spectrophotometric assessment of the consumption of NADH, which serves as a measure that is proportional to the LDH activity. A parameter that is routinely used to simplify the interpretation of the results is the LDH index, representing the ratio of LDH activity in the cell culture medium over the total LDH activity in the cells [9,10,216].

The LDH leakage assay is among the most frequently used procedures to test potential cytotoxic events of a chemical due to its high sensitivity, agility and relatively low-cost [10]. Additional advantages include the possibility of analysing the LDH activity at different time points in a single test (real-time measurement) as well as the high stability of the LDH enzyme when compared with other enzyme-based assays, such as adenylate kinase or glyceraldehyde-3-phosphate dehydrogenase-based assays [9]. Despite the advantages, the presence of certain compounds in the cell culture medium that can alter the LDH activity and stability, such as sodium pyruvate, phenol red or components of the fetal bovine serum [11,12,13].

#### 4.1.2. Calcein-AM Assay

The calcein-acetoxymethyl (calcein-AM) assay is a cytotoxicity method used for the indirect evaluation of the cell plasma membrane integrity by means of assessing the activity of cytoplasmic non-specific esterases [14]. These enzymes, whose activity is limited to cells with an intact membrane, catalyse the conversion of calcein-AM, a permeable, hydrophobic and non-fluorescent compound, into calcein, an insoluble fluorescent green dye [15,16,217]. Thus, this fluorescent signal is proportional to the number of viable cells. Despite the availability of other acetoxymethyl ester derivatives, such as carboxyfluorescein diacetate or bis-carboxyethyl-carboxyfluorescein, calcein-AM is among the most frequently used compounds due to its lower risk of spontaneous leakage upon cell entering [15].

Apart from general advantages, such as ease of use, safety and low-cost, the calcein-AM assay stands out due to its potential application in high-throughput (HTP) strategies, allowing its combination with simultaneous read-outs in a single test run [14,15]. Although extremely valuable, the accuracy of this assay can be compromised due to the transient nature of the calcein-AM signal [16], the difficulties of certain types of adherent cells to uptake the dye or the potential fluorescent signal overlap between calcein and the chemical tested [14,16].

#### 4.1.3. Protease Activity Assay

The protease activity assay is a popular methodology used to indirectly evaluate cell plasma membrane integrity by measuring the activity of conserved and constitutive cellular proteases. The principle of this assay relies on the capability of these enzymes to convert non-colorimetric substrates into coloured products that are quantifiable via fluorescence and/or bioluminescence [17].

Different protease activity approaches have been developed over the last decade, and can be grouped in different categories according to the nature of the substrate used (permeable versus non-permeable) and the localisation of the proteases (intracellular versus extracellular). In this regard, cell membrane permeable substrates, such as glycyl-phenylalanyl-aminofluorocoumarin (GF-AFC), are used to monitor the activity of intracellular proteases, resulting in a signal directly proportional to the number of viable cells [17]. On the other hand, non-permeable cell plasma membrane substrates, such as 2-acetylaminofluorene-aminoluciferin (AAF-aminoluciferin), are commonly used to quantify dead cells by detecting the activity of proteases released into the extracellular environment [218]. In this second approach, cell viability is typically calculated by subtracting the signal of the experimental condition from the signal obtained in a control condition, in which total lysis of the sample is performed [18].

Protease activity-based methods have shown good correlation with other well-established cell viability procedures, such as the ATP content assay or the LDH leakage assay [17,218]. Additionally, the low toxicity of the reagents not only allows for simultaneous combination of this method with other read-outs, but also enables determination of cell viability at different time points in a single test run, making it a popular candidate for HTP strategies. Nevertheless, the presence of proteases in cell culture medium constituents as well as the potential interference of the test chemical with the signal produced by the proteases must be carefully considered [17,18].

#### 4.1.4. Trypan Blue Exclusion Assay

The trypan blue exclusion assay is a versatile staining method commonly used to quantify cell death as well as to count cells prior to in vitro cell seeding [219]. This assay is based on the ability of the trypan blue molecule, a large anionic tetrasulfonated dye, to only penetrate disrupted cell plasma membranes and in turn stain intracellular proteins. As a result, dead or dying cells will be visualised as dark blue swollen spots, whereas viable cells will remain unstained, small and refractive [220]. The number of dead cells can be easily monitored upon light microscopy inspection using a hemocytometer, or automatically using a bench-top automated cell counter [9].

Although being an agile, simple and low-cost in vitro cytotoxicity assay, some considerations should be kept in mind when trypan blue is used, including the intra-operator and inter-operator variability, the dichotomic nature of the results (live versus dead) and the manual labour involved when working with high volumes of samples [19]. Furthermore, it should be stressed that the cytotoxic effect of trypan blue in mammalian cells can cause significant alterations in the sensitivity of the assay. In this respect, optimisation of the dye concentration and the exposure time is strongly encouraged [20,21].

### 4.2. Mitochondrial Functionality Assays

#### 4.2.1. Tetrazolium Salt Assays

The [3-(4,5-dimethylthiazol-2-yl)-2,5-diphenyltetrazolium bromide] (MTT) assay is a multi-step colorimetric method based on the ability of metabolically viable cells to reduce the yellow tetrazolium salt MTT into blue-purple insoluble formazan crystals, which are retained inside the cells. By adding dimethyl sulfoxide (DMSO) or acid/isopropanol solvents, these crystals solubilise and become released in the cell culture medium, allowing their measurement via spectrophotometric techniques. The number of surviving cells is directly proportional to the level of formazan product generated [221]. However, it should be mentioned that the actual reduction of MTT is not only merely the result of the mitochondrial enzyme succinate dehydrogenase activity, but also of other reducing agents and enzymes, located in different cell organelles and subcellular compartments, including the cytosol [222]. Consequently, the MTT assay provides an indicator of overall cell viability and not of mitochondrial activity per se.

Despite being considered as the gold standard in vitro cytotoxicity assay [10,27,28] and being extensively used for testing early cytotoxic events, the MTT assay is not exempted from limitations [22,23]. In this regard, different factors can cause significant deviations in the actual cell viability, including the cell metabolic activity, which is variable along the cell cycle, the different culture phases (stationary versus log phase) and/or the cell type [9], the presence of reductive compounds, such as reduced glutathione, coenzyme A, or even the chemical tested [24,25], and the cytotoxic effect of MTT reagents, which can cause cell damage/apoptosis [26]. In addition, the solubilisation step required for the colorimetric quantification of the formazan product, makes this assay a lytic endpoint methodology, impeding additional read-outs in the sample [9,23].

To counter the limitations related to the low solubility of MTT, a number of alternative water-soluble tetrazolium salts have emerged over the past 2 decades, including, but not limited to, XTT [(2,3-bis(2-methoxy-4-nitro-5-sulphophenyl)-5-carboxanilide-2H-tetrazolium)], MTS (5-(3-carboxymethoxyphenyl)-2-(4,5-dimethylthiazoly)-3-(4-sulfophenyl)-tetrazolium, inner salt), and WST-1 [(4-[3-4-iodophenyl]-2-(4-nitrophenyl)-2H-5-tetrazolio)-1,3-benzene disulfonate)]. Although these compounds are highly stable and sensitive, the selection of an electron-coupling agent, such as phenazine methosulfate, is required to assist and enhance the formation of formazan [10,14,223,224].

#### 4.2.2. Resazurin Reduction Assay

The resazurin (7-hydroxy-3-oxo-3H-phenoxazine 10-oxide) reduction assay is a ubiquitous colorimetric assay used to monitor the reduction capacity of metabolically viable cells. This assay is based on assessing the ability of mitochondrial and cytosolic reductases and diaphorase-like enzymes to reduce the non-fluorescent permeable dye resazurin into the highly fluorescent pink dye resorufin [10]. The amount of resorufin produced, which can be quantified via spectrophotometric and fluorometric techniques, is directly proportional to the number of viable cells [29].

The resazurin reduction assay is agile, easy to perform and can be combined with other in vitro read-outs in a single test set-up, making it eligible for HTP strategies. Additionally, this assay offers a number of advantages, especially compared to tetrazolium-based assays, including higher sensitivity and the possibility of real-time analysis in a single test run [14,27,28,29,30]. Conversely, and although resazurin reduction does not disrupt the mitochondrial respiratory chain, certain cellular alterations, including oxidative stress and/or cell death, have been reported in some cell lines [27,31]. An additional disadvantage of this method relates to the time required to generate an adequate signal, which has to be optimised for each cell type [27], as well as the potential interference of serum proteins with the signal produced by resorufin [32].

#### 4.2.3. ATP Content Assay

The ATP content assay is based on monitoring intracellular ATP levels as an indirect biomarker of cellular functional integrity [33,225]. Several methods have been described for measuring ATP content, with the bioluminescent luciferin-luciferase among the most routinely used assays due to its particular sensitivity and reliability [10,33,34]. The principle of this assay is based on the ability of the enzyme luciferase to catalyse the oxidation of luciferin, a reaction that requires ATP consumption and that results in the emission of a quantifiable flash yellow-green signal. The amount of ATP present in the cell, which reflects cellular viability, is directly proportional to the amount of light that is generated [225].

The ATP content assay offers a number of advantages, especially compared to tetrazolium-based assays, including better sensitivity and reproducibility, higher stability of the signal (up to 5 h), lower or almost absent levels of background noise, possibility of monitoring lower cell numbers, and absence of incubation steps to convert the substrate into a coloured product. It should be stressed that ATP levels are a critical cellular parameter that are drastically depleted when cell damage occurs, avoiding the need of long exposure periods with the tested chemical. This makes the ATP content in vitro assay a rapid strategy to assess cell viability [34,35,36]. An even more important consideration is the potential use of this assay to evaluate 3D culture platforms, which is highly relevant due to the growing numbers of 3D in vitro models that are being developed to study liver diseases [37,38].

Despite being extremely valuable, certain limitations of this in vitro assay should be kept in mind, including the alterations of the luciferase activity by the test chemical and/or the culture conditions, the high cost and the additional lysis step required for the extraction of intracellular ATP. In this regard, the lytic nature of the assay not only makes it an apical approach, but also leads to the release of ATPases that can degrade ATP molecules and jeopardise the results of the assay [10,27,33].

#### 4.2.4. Mitochondrial Membrane Potential Evaluation: Fluorescent Probe-Based Assays

Mitochondrial membrane potential assays are based on evaluating alterations in the mitochondrial membrane potential as a reliable indicator of mitochondrial dysfunction, which has been linked to cell death [39,226,227]. This physiological parameter can be defined as the difference in the potential of the mitochondrial membrane derived from the asymmetrical distribution of protons and other ions on both sides of the inner membrane of this organelle [197,228].

Several tools and techniques have emerged over the past 2 decades to quantify changes in mitochondrial membrane potential, with organic fluorescent membrane-permeable probes being the most frequently used methods. These methods are based on the tendency of lypophylic cationic dyes to penetrate and accumulate in the matrix of active mitochondria, as a result of their negative membrane potential. In turn, this accumulation leads to a quantifiable fluorescent signal, which is proportional to the mitochondrial membrane potential status. More polarized mitochondria will accumulate more cationic dye, resulting in a higher fluorescence signal that can be measured using a flow cytometer, a fluorescence microscope or a plate reader [40,41].

Three main families of fluorescent probes have been defined according to their detection method [42]. Monochromatic and ratiometric fluorescence probes are most popular and include, but are not limited to, the commercially available probes Rhodamine 123 (Rhod 123) [229], 3,3′-dihexyloxacarbocyanine iodide [DiOC_6_(3)] [230], tetramethylrhodamine methyl ester perchlorate (TMRM) or tetramethylrhodamine ethyl ester perchlorate (TMRE) [40,231], the 5,5′,6,6′-tetrachloro-1,1′,3,3′-tetraethylbenzimidazolcarbocyanine iodide (JC-1) [232] and the mitochondrial membrane potential indicator (mMPI) [41].

Although the use of fluorescent probes to evaluate the mitochondrial membrane potential is abundant, it still carries some limitations, such as the sensitivity of the approach, which is not enough to detect subtle aberrant changes in MMP, the cytotoxicity of the dye per se, which may alter the normal functioning of mitochondria/cells, the non-specificity of certain probes, which may bind to membrane components other than mitochondria, and the low water solubility of certain probes, such as JC-1, which compromises in vitro application. Therefore, the selection of a specific dye and the interpretation of the results should be carefully performed. Linked to this, the use of fluorescent probes should be accompanied by appropriate pharmacologic controls as well as complementary dyes, which allows to increase the robustness of the results [39,40,41,42,43].

### 4.3. Oxidative Stress Assays

#### 4.3.1. Intracellular ROS Quantification: DCFH_2_-DA Fluorescence Probe-Based Assay

The 2′,7′-dichlorodihydrofluorescein diacetate (DCFH_2_-DA) assay is one of the most widely used fluorescent probe-based methods to evaluate intracellular reactive oxygen species (ROS) levels. This assay is based on the capacity of the lipophilic probe DCFH_2_-DA to pass through the cell plasma membrane and to become a non-fluorescent non-permeable product when hydrolysed by cellular esterases. This resulting molecule is subsequently oxidised by cellular ROS, leading to a highly fluorescent molecule (DHFH_2_), whose signal intensity is proportional to the amount of ROS present in the cell. This signal can be measured by using a flow cytometer, a fluorescence microscope or a plate reader [44,45]. Although DCFH_2_-DA is the preferred method of choice for monitoring intracellular ROS levels, other fluorescent probes based on the same principle are also available, such as dihydrorhodamine 123 [233].

A major concern of this method is the specificity of the probe, which reacts not only with different ROS species, such as hydroxyl or peroxyl radicals, but also with certain cellular components, including cellular peroxidases, cytochrome C or cellular antioxidants [46,47]. Because of this, the DCFH_2_-DA assay is an indicator of the overall cellular oxidative state and not of levels of specific ROS species [44,45,48]. Furthermore, artificial amplification of the fluorescence signal intensity due to intermediate oxidative products of the probe, such as superoxide radicals, must be considered as well [49]. Moreover, it should be stressed that the low-charged state of DCFH_2_ can lead to extracellular leakage of this molecule. Alternative non-fluorescent derivatives of DCFH_2_-DA displaying better cellular retention have been developed and are commercially available [50].

#### 4.3.2. Intracellular/Mitochondrial Superoxide Quantification: DHE/Mito-HE Fluorescence Probe-Based Assays

The dihydroethidium (DHE) fluorescence assay is a popular fluorescent probe-based method used to monitor intracellular ROS species, in particular superoxide radicals. The DHE assay is based on the capacity of the membrane permeable non-fluorescent probe DHE to be oxidised by intracellular superoxide radicals, resulting in the formation of a red fluorescent product (hydroxyethidium, 2-OH-E^+^), whose signal intensity is proportional to the amount of superoxide radicals present in the cell. This signal can be measured by using a flow cytometer, a fluorescence microscope or a plate reader [53].

Although extensively used to quantify superoxide radicals, it should be stressed that DHE can also react with other oxidants rather than superoxide radicals, generating a non-specific signal. The resulting products, in particular ethidium, exhibit a fluorescence spectrum very similar to 2-OH-E^+^ and can potentially cause a significant deviation in actual superoxide radical levels [53,54]. In this regard, some groups have indicated a limited quantitative value of this assay when using standard fluorescence devices, such as plate readers or microscopes, even when combined with precise filters and carefully selected excitation wavelengths [54,234]. In order to overcome this issue, certain strategies have been implemented to directly quantify the formation of 2-OH-E^+^, such as the use of high-performance liquid chromatography (HPLC) or liquid chromatography-mass spectrometry (LC–MS) [235,236].

In recent years, a modified cationic conjugated form of DHE has emerged to specifically measure mitochondrial superoxide species. This probe is known as Mito-HE and follows the same mechanistic principle as DHE. The positive charge of the probe allows to rapidly accumulate in the mitochondria, where it reacts with superoxide species, thereby producing a red fluorescent signal quantifiable using a flow cytometer, a fluorescence microscope or a plate reader [55,56]. As mentioned, and although mitochondrial superoxide species are the major ROS oxidisers of Mito-HE in the mitochondrial compartment, other mitochondrial one-electron oxidants, such as cytochrome C peroxidase, and/or hydrogen peroxide can cause oxidation of the probe [54,57]. Critical factors to improve the accuracy of Mito-HE-based assays include optimisation of probe concentrations together with the use of superoxide-specific scavengers, such as superoxide dismutase mimetic hydrophilic carbon clusters [51,52].

#### 4.3.3. Lipid Peroxidation: MDA/TBARS Assay

The malondialdehyde (MDA) assay is a widely used method to quantify the amount of MDA, one of the major products derived from oxidation of the double bonds of polyunsaturated FAs. The evaluation of MDA concentrations allows to indirectly determine cellular levels of ROS based on assessing oxidative damage caused by these radicals to lipids [237,238].

Several methods and techniques have been developed over the past decades for measuring MDA levels, including gas chromatography-mass spectrometry [239], HPLC [240], and LC-MS [241]. Nonetheless, the colorimetric/fluorimetric assay based on the use of thiobarbituric acid (TBA) is one of the most commonly used approaches. This method relies on the quantification of a strong ultraviolet-visible compound produced upon reaction of TBA with MDA at high temperatures and low pH, which is directly proportional to the amount of MDA present in the sample [242,243].

Although the use of the TBA method is very popular due to its agility (2 h), simplicity and relatively low cost, some important caveats have been reported [58,59]. Firstly, TBA can react with numerous chemically reactive carbonyl-containing organic molecules other than lipids, causing overestimation of the amount of MDA [60,61]. Therefore, the TBA assay is routinely denoted as TBARS (thiobarbituric acid reactive substances) and the results are expressed as “MDA equivalents” to emphasize that results are not exclusive for MDA [61,237]. Secondly, the harsh conditions required for this assay (high temperature and low pH) can cause artificial peroxidation of the sample, jeopardising the interpretation of the results. Thirdly, the detection limit of the TBA assay is relatively low (1.1 μM), compromising the quantification when samples have low levels of MDA [58,62].

#### 4.3.4. Antioxidant Status Assays: Evaluation of Enzymatic Antioxidant Activity

The monitoring of critical antioxidant enzymes and low molecular weight cofactors implicated in the redox homeostasis system of the cell is a popular method to evaluate levels of cellular oxidative stress. Typically, the evaluated enzymes include superoxide dismutase (SOD), which catalyses the dismutation of the superoxide anion to hydrogen peroxide and molecular oxygen, catalase, which catalyses the neutralization of hydrogen peroxide to water, and glutathione peroxidase (GPx), which catalyses the reduction of both hydrogen and lipid peroxides to water and lipid alcohols, respectively, via the oxidation of reduced glutathione [63,244].

A number of methodologies have been implemented to measure the activity of these enzymes [63,64,65], including colorimetric-based, electrophoresis-based [245] and histology-based methods [246]. Colorimetric-based assays are grouped in 2 categories, namely direct or indirect methods. The former includes methods based on assessing enzymatic activity, such as the reduction of hydrogen peroxide for catalase activity quantification or the autooxidation of 5,6,6a,11b-tetrahydro-3,9,10-trihydrobenzo[c]fluorine (BXT-01050) for SOD activity quantification [247,248]. The latter methods determine enzymatic activity via secondary reactions, such as the combination of the xanthine-xanthine oxidase cytochrome C with the reduction of tetrazolium salts for SOD activity quantification [249,250].

Colorimetric assays are routine constituents of advanced batteries for quantifying antioxidant enzymatic activity due to the high specificity and the quantitative nature of the results [63,64,65]. Yet, several limitations are encountered, such as the higher amounts of the test material required compared to electrophoresis-based methods or the lytic and hence disruptive nature of these assays. In this regard, the lysis step impedes the quantification when evaluating mixed cell populations and does not allow gathering information about the cellular localisation of the enzyme. Moreover, the existence of different antioxidant enzyme isoforms should not be disregarded when analysing and interpreting the results. Thus, additional control conditions are strongly encouraged for creating specific isoform signatures [63,64].

### 4.4. Cell Death Assays

#### 4.4.1. Annexin V Staining Assay

The annexin V staining assay has been widely applied to detect early phase apoptosis due to the high affinity of annexin V, a calcium-dependent phospholipid binding protein, for phosphatidylserine (PS) exposed at the cell plasma membrane surface during apoptosis [66,251]. Under normal cell conditions, the phospholipid PS is localised on the inner leaflet of the plasma membrane. However, during apoptosis, a redistribution between the layers of the membrane leads to PS externalization (“flip-flop”), which then serves as an “eat-me” signal for phagocytes [252,253]. Such exposure of PS on the outer leaflet of cell plasma membrane can be detected by fluorescently labelled PS-binding proteins, including annexin V, enabling the quantification of apoptotic cells through flow cytometry and fluorescence microscopy [67,68].

The annexin V protein can also pass through compromised cell plasma membranes of dead cells and bind to intracellular PS. Therefore, the combination of annexin V staining with cell-impermeant dyes, such as propidium iodide (PI), is recommended to exclude false positives [69]. Cells with compromised cell plasma membranes will be stained with annexin V and PI and, consequently, cannot be considered as apoptotic. On the other hand, cells only positive for annexin V suggest an apoptotic state [70,71].

The annexin V staining assay allows non-destructive detection of apoptosis with high sensitivity by identifying externalized PS. Interestingly, this method is not toxic, and its application can be extended to the tissue and even whole-body level [72]. However, slow formation of annexin V-PS high affinity complexes impairs the detection of the earliest step of PS exposure. Additionally, unbound labelled annexin V may produce a strong background signal, requiring additional washing steps. Considering that annexin V is a calcium-dependent phospholipid binding protein, the need for calcium ions in mM concentrations may limit its practical application [66].

#### 4.4.2. PI dye Uptake Assay

PI is a membrane non-permeable intercalating agent commonly used for in vitro cell death assessment. PI is excluded by viable cells with intact cell plasma membranes, but invades damaged or dead cells [220,254,255]. Upon cellular uptake of PI, its fluorescence intensity increases up to 10-fold after DNA binding, thereby emitting red light and allowing to distinguish live from dead cells [220,256]. Dead cells can be quantified using fluorescence microscopy and flow cytometry. This is a versatile and low-cost method suitable for fresh, relatively homogeneous samples and for screening purposes in HTP strategies [73]. PI is commonly used in combination with the annexin V for cell death sorting [69]. However, some critical steps during the PI staining procedure, such as dye incubation time, washing buffers and dye concentrations, may increase the occurrence of false PI positive cells. When working with PI, care must be taken, since the dye is mutagenic, requiring careful handling as well as proper waste disposition [74].

#### 4.4.3. Caspase Activity Assays

Several methods have been developed to detect the activation of caspases using different approaches, which comprise in vitro enzyme assays, flow cytometry, fluorescent and light microscopy and enzyme-linked immunosorbent assay (ELISA) methods [257]. Quantification of caspase activity using in vitro enzyme assays is based on cleavage of a substrate by caspase 3. These methods are agile and allow consistent quantification of caspase activity [80]. However, the substrate is not entirely specific for a particular caspase. Thus, the use of more than one method is recommended, such as immunoblotting, to confirm specific caspase activation. Additionally, these assays require cell lysis and, therefore multiplexing with other in vitro assays is impaired [75].

Activation of caspases can be also studied using fluorescent-labelled inhibitors that bind covalently to individual caspase active centers, which are known as fluorochrome-labelled inhibitors of caspases (FLICA) [76,77,258]. Adding FLICA to live in vitro or in vivo cells allows rapid uptake of these reagents with subsequent covalent binding to caspase active centers in cells undergoing apoptosis, which can be measured via flow and laser scanning cytometry. This method is relatively non-toxic and can easily be combined with other markers. Thus, combination with PI enables to evaluate cell plasma membrane integrity. Mitochondrial membrane potential probes are another example of markers that can be combined to caspase activity assay. These probes allow to discriminate dissipation of the inner mitochondrial membrane potential from activation of the caspase enzyme cascade [77,78]. Additionally, FLICA has proven to be a reproducible and sensitive marker for apoptotic cell death. It should, however, be emphasized that FLICA does not have specificity for a given caspase [76,79].

#### 4.4.4. TUNEL Assay

The terminal deoxynUcleotidyl transferase dUTP Nick End Labelling (TUNEL) assay is a method commonly used to detect DNA fragmentation in apoptotic cells. This assay detects the 3′ end of DNA fragments generated by apoptotic cells by replacing some of the DNA fragmented nucleotides with labelled nucleotide analogues. The attachment of labelled 2′-deoxyuridine 5′-triphosphate (dUTPs) nucleotides to the hydroxyl end of DNA strand breaks is catalysed in particular by the endonuclease terminal deoxynucleotidyl transferase (TdT). The labelled-DNA sequence is used as a probe and can be detected via light microscopy, fluorescence microscopy or flow cytometry [81,82]. The TUNEL assay is a very sensitive and readily applicable method [68,80,83]. Another advantage is the detection of DNA strand breaks occurring in the early stage of apoptosis, prior occurrence of morphological changes. This method can be combined with immunofluorescent labelling in order to increase information content [82]. However, the assay is expensive, time-consuming and subject to false positive results from necrotic cells, cells in DNA repair or gene transcription [83,84].

### 4.5. Miscellaneous Assays: Neutral Red Uptake

The neutral red uptake (NRU) is a colorimetric-based method commonly used to monitor lysosomal integrity as an indirect marker of the cellular viability. This assay is based on the ability of living cells to incorporate and accumulate the 3-amino-m-dimethylamino-2-methyl-phenazine dye inside their lysosomes [85,86,259]. Under physiological conditions, neutral red penetrates cell plasma membranes and migrates towards the lysosomes, where it accumulates due to the low pH [85,259]. Alterations of the cell surface or the lysosomal membrane upon cellular injury led to loss of the dye [260]. The amount of neutral red retained in the lysosomes is directly proportional to the number of viable cells, and can be quantified via spectrophotometric techniques.

The NRU assay is a highly versatile cytotoxicity assay, which not only allows for quantification of cell viability, but also permits measurement of cell replication, cytostatic effects or cell death, depending on the cell seeding density [85,87]. This, linked to the agility and possibility of automation in HTP strategies [88,89,90], has led to the establishment of the NRU assay as the standard procedure of European regulatory agencies to evaluate cellular damage caused by diverse types of chemicals [261,262]. Additionally, when compared with other well-established cytotoxicity methods, such as tetrazolium salts-based or LDH leakage assays, the NRU assay has proven more sensitive, stable and low-cost [91,92].

Although extremely valuable and robust, several parameters, such as dye concentration or incubation times as well as pH and temperature, should be optimised and tightly controlled in order to avoid a significant deviation in the actual cell viability read-out [93]. Furthermore, it should be stressed that certain drugs and chemicals, including chloroquine or surfactants, can have a localised effect on lysosomes [94,95] or can induce irreversible precipitation of the dye [263]. As a consequence, the type of chemical tested by this assay should be carefully selected. Finally, the lytic terminal nature of the NRU assay impeding additional read-outs in the sample cannot be obviated [85,86].

## 5. Mechanisms of Liver-Specific Toxicity

**Figure 3:**
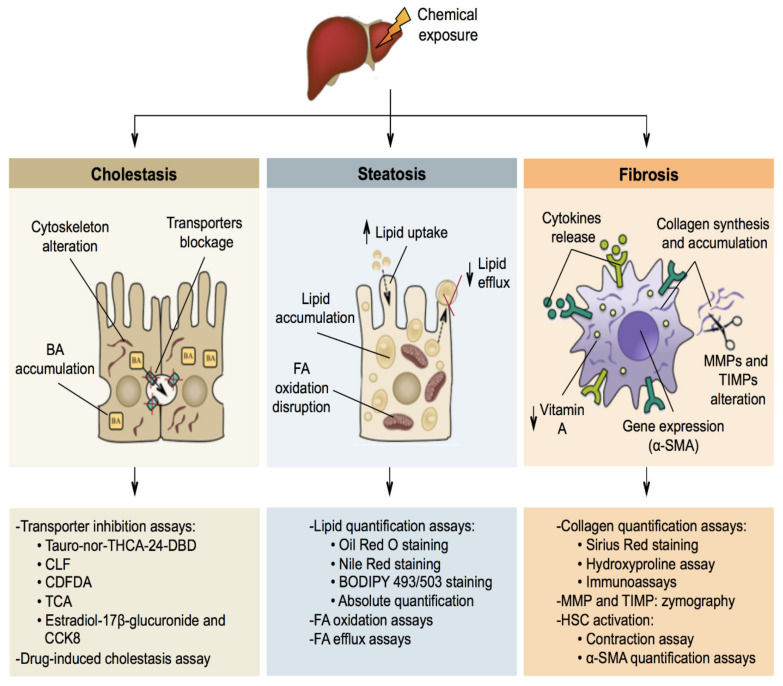
Schematic representation of the 3 main types of liver-specific toxicity and corresponding in vitro assays. BA: bile acid; BODIPY 493/503: 4,4-difluoro-1,3,5,7,8-pentamethyl-4-bora-3a,4a-diaza-s-indacene 495/503; CCK8: cholecystokinin-octapeptide; CDFDA: 5(6)-carboxy-2′,7′-dichlorofluorescein diacetate; CLF: cholyl-lysyl-fluorescein; FA: fatty acid; MMPs: matrix metalloproteinases; HSC: hepatic stellate cell; Tauro-nor-THCA-24-DBD: tauro-nor-*N*-(24-[7-(4-*N*,*N*-dimethylaminosulfonyl-2,1,3-benzoxadiazole)]-amino-3α,7α,12α-trihydroxy-27-nor-5β-cholestan-26-oyl)-2′-aminoethanesulfonate; TCA: taurocholate; TIMPs: tissue inhibitor of metalloproteinases; α-SMA: alpha-smooth muscle actin.

Liver toxicity or hepatotoxicity refers to injury to the liver as a result of damaging or destructive agents, mainly chemicals. In most cases, the hepatocytes are the main targets of hepatotoxic chemicals, yet in some forms of liver toxicity, other liver cell types are involved as well. Depending on the duration between the first contact with the noxious chemical and the manifestation of the adverse liver effects, a distinction can be made between acute (hours/days) and chronic (weeks/months/years) hepatotoxicity [264]. Hepatotoxicity presents itself clinically in a number of ways, including, but not limited to, cholestasis, steatosis and fibrosis (Figure 3).

### 5.1. Cholestasis

The formation of BAs in hepatocytes depends on osmotic and active transport of BAs into the canalicular lumen followed by the passive flux of water through tight junctions [265,266,267]. Several hepatobiliary transporter proteins, located at the sinusoidal and canalicular membrane poles, are involved in this process and play a key role in the regulation of intrahepatic and systemic BA levels. Among those is the bile salt export pump (BSEP), which conveys bile salts from hepatocytes into the bile canaliculi [266,267,268,269,270]. Cholestasis denotes any situation of impaired bile secretion concomitant with BA accumulation in the liver and/or in the systemic circulation. A difference is made between intrahepatic and extrahepatic cholestasis, depending on the location of the blockage. Hepatocytes typically adopt a brownish appearance in cholestasis, which reflects BA accumulation [102,269,271,272]. Furthermore, canalicular bile plugs between hepatocytes or within bile ducts may occur. These bile plugs increase pressure, which causes rupture and spilling of BAs into surrounding tissue. In turn, this activates necrosis and inflammation [269,273]. Chemicals, mainly drugs, can induce cholestasis in many ways, such as through internalization or inhibition of the BSEP, modulation of cell plasma membrane fluidity, impairment of tight junctions and alteration of the cytoskeleton [102,272].

### 5.2. Steatosis

FAs can be synthesised and esterified from acetylcoenzyme A and glycerol by hepatocytes. FAs can also be taken up as chylomicrons from the blood. They are catabolized in hepatocytes through mitochondrial β-oxidation. Nevertheless, FAs are predominantly used as a source for the synthesis of lipids, including triglycerides [274]. Steatosis refers to the aberrant retention of lipids, mainly triglycerides, within hepatocytes, and results from impaired synthesis and elimination of triglycerides. Steatosis can progress towards steatohepatitis, which is featured by pronounced inflammation [274,275,276]. Steatosis can result from several cellular mechanisms, including alterations in FA oxidation (FAO), with disruption of mitochondrial-mediated β-oxidative metabolism, changes in lipid efflux, with a decrease in the secretion of very low-density lipoproteins (VLDL), and/or modifications in lipid uptake [143,277,278,279].

### 5.3. Fibrosis

Fibrosis basically is a wound-healing response to chronic liver injury, whereby quiescent HSC turn into contractile, proliferative and fibrogenic myofibroblasts-like cells [280]. Activation of HSCs occurs in 2 steps, namely initiation and perpetuation. Initiation is triggered by soluble factors, such as apoptotic bodies, as well as by paracrine signals from hepatocytes, sinusoidal endothelial cells and Kupffer cells. It involves loss of the cellular vitamin A content, increased expression of fibroblast-related genes, such as α-smooth muscle actin (α-SMA), sensitisation to several mediators, including oxidative species and growth factors, and major changes in the ECM homeostasis. The remodelling of the ECM is characterised by accumulation of collagen type I and III as well as by an imbalance in matrix metalloproteinases (MMPs) and tissue inhibitor of metalloproteinases (TIMPs) levels. In the perpetuation step, activated HSCs acquire cell contractility capacity and massive cytokine release is observed, leading to the activation of Kupffer cells and the deposition of ECM compounds into the space of Disse [187,188,280,281]. This so-called capillarization event results in the disappearance of hepatocyte microvilli and the loss of sinusoidal endothelial cell fenestrae. The most progressive form of fibrosis is called cirrhosis, which is considered an irreversible event [282,283].

## 6. Liver-Specific Toxicity In Vitro Methods

### 6.1. Cholestasis Assays

#### 6.1.1. Transporter Inhibition Assays

Hepatobiliary transporters are essential for maintaining BA homeostasis. They are mainly involved in preserving bile formation and the enterohepatic circulation of BAs. Therefore, they constitute key players of intrahepatic and systemic BA levels [270]. Reduced activity of hepatobiliary transporters can lead to hepatic accumulation of BAs and, consequently, result in hepatotoxicity. Considering that BAs are a sensitive index of liver injury, the assessment of hepatobiliary transporter activity using specific substrates is a possible read-out for testing the cholestasis-inducing potential of chemicals [284]. A plethora of substrates is used for evaluating the activity of BSEP, multidrug resistance-associated protein (MRP) 2/3, sodium taurocholate co-transporting polypeptide (NTCP) and organic anion transporting polypeptides (OATP) 1B1/1B3.

##### Tauro-nor-THCA-24-DBD

Measurement of BSEP inhibition relies on the microscopical monitoring of the accumulation of fluorescent BSEP substrates, such as *N*-(24-[7-(4-*N*,*N*-dimethylaminosulfonyl-2,1,3-benzoxadiazole)]-amino-3α,7α,12α-trihydroxy-27-nor-5β-cholestan-26-oyl)-2′-aminoethanesulfonate (tauro-nor-THCA-24-DBD), in the canalicular network. Although primarily visual, the outcome of this assay can be quantified using spectrophotometric techniques [285]. Most BSEP substrates cannot undergo cellular translocation without the support of an uptake transporter protein, in particular NTCP. In fact, several chemicals known to suppress BSEP activity also affect NTCP. This must be considered, as it may complicate the interpretation of the experimental results. Taking into account the role of NTCP in tauro-nor-THCA-24-DBD uptake Iin hepatocytes, the application of this probe has also been extended to study the effects of chemicals on NTCP activity [96].

##### CLF

Cholyl-lysyl-fluorescein (CLF) is a fluorescent-labelled BA analogue commonly used as indicator of bile accumulation [97]. CLF has similarities in biliary output and hepatic extraction with the naturally occurring BA cholylglycine. As a consequence, CLF has been used to explore biliary excretion both in vitro and in vivo [286,287]. CLF efflux inhibition also allows for direct visualization of CLF secretion into bile canaliculi by BSEP. Furthermore, it enables the quantification of inhibitory activity of this process by a variety of compounds. Although BSEP has been reported to be the main CLF efflux transporter, MRP2 has also been recognised to mediate biliary excretion of this substrate [98,287]. The CLF efflux assay has the ability to predict the cholestatic potential of chemicals and is suitable for HTP screening [98,288]. Additionally, this assay enables multiplexing with other fluorescent microscopy-based toxicity assays. Although the CLF efflux assay is an agile and low-cost procedure, it is prone to artefacts and compound interference [98,289].

##### CDFDA

The hepatobiliary transporters MRP2/3 can be monitored using the canalicular marker 5(6)-carboxy-2′,7′-dichlorofluorescein diacetate (CDFDA), which readily permeates into hepatocytes and subsequently undergoes hydrolysis by intracellular esterases. The fluorescent metabolite CDF is secreted into the bile canaliculi by cell plasma membrane transporters, particularly MRP2 [290,291]. This is a robust assay and a suitable tool for investigation of the inhibitory potential of compounds and MRP2-mediated interactions [99].

##### TCA

Similar to the BA taurocholate (TCA), the hepatic uptake of radiolabelled or fluorescent-labelled TCA is predominantly mediated by NTCP [292,293,294]. Efflux of labelled TCA substrates by BSEP can be also quantified. The uptake assay using fluorescent-labelled TCA enables straightforward quantification of NTCP uptake activity by fluorescence microscopy. Although the radiolabelled-TCA substrate is highly sensitive and widely used [100,295,296,297], it raises safety concerns and the need to deal with waste disposal.

##### Estradiol-17β-Glucuronide and CCK8

Estradiol-17β-glucuronide and cholecystokinin-octapeptide (CCK8) can be used as selective probe substrates for evaluating OATP 1B1 [298,299,300] and OATP 1B3 [101,301] transporter activities, respectively. Measurement of OATP1B1/1B3 inhibition can also rely on the uptake of other specific substrates, such as zombie violet, live/dead green, cascade blue hydrazide, Alexa Fluor 405 succinimidyl ester [302], estrone-3-sulfate [101,303], statins [304] and sodium-fluorescein [305]. Optimisation of fluorescence-based in vitro transporter assays using sodium-fluorescein as probe substrate has allowed for HTP screening of the inhibitory potential of chemicals [305].

#### 6.1.2. Drug-Induced Cholestasis Assay

The drug-induced cholestasis (DIC) assay has been developed to distinguish cholestatic compounds from hepatotoxic non-cholestatic and non-hepatotoxic compounds based on their potential to modulate BA disposition in a sandwich-cultured human hepatocytes in vitro model. This assay enables early assessment and prediction of an increased DIC risk [103] as well as the determination of the drug-induced cholestatic index (DICI). The DICI relies on the ratio of urea produced by the sandwich-cultured human hepatocytes exposed to test compound in the absence or presence of a BA mixture. A DICI cut-off value of 0.78 is used to correlate obtained in vitro results with an in vivo safety margin [103]. The use of sandwich-cultured human hepatocytes has the advantage of preserving both phenotype and liver-specific functions for prolonged periods of time. Alterations in intracellular BA levels in this in vitro model may also provide useful mechanistic information on cholestatic compounds [102]. However, this model may not provide accurate assessment of long-term toxicity due to time-dependent downregulation of hepatic transporters and enzymes [104].

### 6.2. Steatosis Assays

#### 6.2.1. Lipid Quantification Assays

##### Oil Red O Staining

The 1-(2,5-dimethyl-4-(2,5-dimethylphenyl) phenyldiazenyl) azonapthalen-2-ol, Sudan Red 5B or Oil Red O (ORO) is a lysochrome diazo dye frequently used to determine the levels and the intracellular localisation of hydrophobic and neutral lipids, such as triglycerides, diacylglycerols and cholesterol esters [306,307]. ORO staining has been described as one of the most accurate procedures for evaluating liver steatosis in vitro [105,308,309,310]. The principle of this staining is based on the poor solubility of ORO when prepared in a water-based solvent and its affinity for hydrophobic substrates. Hence, after adding ORO to the sample, the dye moves from the solvent towards the hydrophobic and neutral lipids, staining them with an orange-red coloration [106,306,307]. Due to the molecular nature of the dye, it can be evaluated using both light and fluorescence microscopy. The latter has shown to improve the signal and quantification of the lipid droplets when using appropriated filters [107]. Additionally, ORO levels can be measured using a plate reader. Nevertheless, it requires an additional step of dye extraction from the sample [105,108].

Important advantages of the ORO staining method include the relative low cost of the technique, the basic laboratory equipment required and the possibility of quantifying the signal using computed-based software [106,107,109]. Furthermore, this assay can be combined with other stains, such as 4′,6-diamidino-2-fenilindol (DAPI) [107] or hematoxylin and eosin [110], enabling in-depth assessments. Although extensively used, several considerations should be kept in mind when ORO staining is performed. Thus, all hydrophobic and neutral lipids present in the sample are stained, impeding the possibility of distinguishing particular lipid species [111]. The use of alcohol-based fixatives and paraffin-embedded procedures should be avoided in order not to jeopardise the lipid levels or the lipid droplet morphology, especially when working with 3D in vitro models [112,113]. Finally, ORO working solutions must be prepared ex tempore and filtrated before use, and the stability of the signal can be lost after 3 days of staining [106].

Other sudan lysochrome diazo dyes also used to evaluate steatosis include the 1-(4-(phenylazo)phenylazo)-2-naphthol (Sudan III), the 1-(2-methyl-4-(2-methylphenylazo)phenylazo)-2-naphthol (Sudan IV) and the 2,3-dihydro-2,2-dimethyl-6-((4-(phenylazo)-1 naphthalenyl)azo)-1H-pyrimidine (Sudan Black B) [311,312,313].

##### Nile Red Staining

The 9-diethylamino-5H-benzo[a]phenoxazine-5-one, or Nile Red (NR), is an uncharged benzophenoxazone metachromatic fluorescent dye typically used to localise and quantify cytoplasmic lipids. In particular, it allows detection of neutral lipid droplets within cells, such as triacylglycerols or cholesterol esters. This dye was initially described as a minor component of another lipid staining, namely Nile blue A, where it triggered the metachromatic pink colouring of neutral lipids [114]. The NR staining method is based on 2 properties of the dye. Thus, it is hydrophobic and therefore only soluble in organic solvents and lipids, and it has the particularity of varying the maximum fluorescence colour emission spectra depending on the polarity of its environment. Accordingly, when excited, NR is almost non-fluorescent in water, whereas it emits a yellow-orange signal in non-polar environments that shifts to the red spectra when in contact with semi-polar environments, allowing the detection of neutral lipids and phospholipids, respectively. The fluorescent signal can be measured by using a flow cytometer, a fluorescence microscope or a plate reader.

The NR staining is a highly versatile technique frequently used for evaluating lipid content in in vitro models of liver steatosis [115,116,117,118]. This staining has a number of advantages, including higher sensitivity when compared to other stains, such as ORO [119], potential application in live cells, since no fixation is required [114,120], preparation of the dye solution in aqueous medium, not requiring the use of organic solvents and therefore minimizing the risk of dissolving the targeted lipids, and implementation in HTP strategies [116,117,121,122]. An even more important advantage is the solvatochromic property of NR that allows for detection and identification of different lipid components in the sample of interest according to their different fluorescent signal, increasing the accuracy of the assessment [121,123]. Nevertheless, the broad absorption and emission spectra of this dye, linked to its solvatochromic property, can also result in the staining of non-specific components with hydrophobic domains as well as in cross-talk in the red channel, jeopardising determination of the lipid content and making this staining unsuitable for multicolour imaging, respectively [124,125].

##### BODIPY 493/503 staining

The 4,4-difluoro-1,3,5,7,8-pentamethyl-4-bora-3a,4a-diaza-s-indacene 495/503, boron-dipyrromethene 495/503 (BODIPY 493/503), is a cell-permeable lipophilic fluorescent dye belonging to the BODIPY fluorophore family [314], frequently used to localise and quantify cytoplasmic lipids. In particular, BODIPY 493/503 allows a selective detection of neutral lipids contained within lipid droplets [126,127,128,315,316]. The BODIPY 495/503 staining method is based on the capacity of the dye to emit a green fluorescent signal exclusively when in contact with non-polar environments, such as neutral lipids. This signal can be quantified via fluorescence microscopy and/or flow cytometry [126,127,128]. The narrow emission spectrum of BODIPY 495/503 [129,315] makes it an ideal tool for simultaneous detection approaches, such as dual localisation experiments [126,130]. Despite the advantages, the small Stockes shift should be optimised, since this could cause cross-talk between the excitation source and the fluorescence emission when filters are not carefully selected [125].

Some remarkable advantages of the BODIPY 494/503 staining method include its agility, linked to the fast cell penetration of the dye [131], the potential evaluation of both live and fixed cells [130], and the stability of the dye for long-term storage periods, thereby reducing the workload associated with the ex tempore preparation of the dye. Although valuable, certain practical aspects linked to the nature of the dye should considered when performing this staining, especially in comparison with similar dyes, such as NR, including the higher background noise due to its non-fluorogenic nature, lower levels of fluorescence intensity and faster photobleaching [129,132,133].

##### Absolute Lipid Quantification Assays

The absolute quantification of total and/or small fractions of lipids contained in the cell is a frequently used method for evaluation of lipid levels present in in vitro liver steatosis models [116,317,318,319]. Cellular lipid quantification is a multi-step procedure, which starts with a cellular lysis step followed by the extraction of the lipids, leading to their quantification in the last step. The extraction procedure is a critical step in the workflow of lipid analysis, since it purifies the lipids and removes potentially interfering substances, such as proteins, carbohydrates and other polar metabolites. For this purpose, the Folch-Bligh and the Dyer methods and their respective variants are among the most frequently used protocols [320,321,322,323,324,325,326]. Both methods are based on the use of chloroform and methanol to extract and dissolve the lipids, and the subsequent addition of water to purify and separate the different species of lipids present in the sample.

Another key point in the workflow of lipid analysis is the system used to quantify the lipids. Several methods and techniques have been adapted for this purpose, including, -HPLC, LC-MS or fluorimetric/colorimetric-based methods [134,135,136]. In this regard, although sensitive and valuable, LC-MS or HPLC require the use of expensive infrastructure, trained personnel and time-consuming and complex procedures, compromising implementation of cellular lipid quantification in routinely batteries for testing steatosis in vitro [134,136]. On the other hand, and although not always with high sensitivity, the use of fluorimetric/colorimetric-based methods has emerged as an alternative and attractive approach due to their simplified sample handling, low cost and potential application in HTP strategies [135]. Several commercial assays are currently available for quantifying both total lipids and certain lipid fractions, including cholesterol and triglycerides. Among those, the sulfo-phospho-vanillin method is the most frequently used one [137,138,139]. It should be stressed that these methods allow the absolute quantification of lipid content, but not the assessment of their subcellular localisation. For this reason, these methodologies should be combined with other stain-based read-outs, such as ORO, NR or BODIPY 495/403, in order to create high accurate integrated approaches for in vitro testing of liver steatosis.

#### 6.2.2. FAO Assays

The analysis of FAO, a mitochondrial β-oxidation mechanism used by hepatocytes to produce energy by means of transforming FAs to acetylcoenzyme A with concomitant production of NADH [327], has been recently proposed as a complementary method to evaluate and monitor the development of liver steatosis in vitro [143,276,279]. A number of methodologies have been introduced to measure FAO, including indirect assays based on the quantification of subproducts resulting from FAO reactions [63,64,65]. In this regard, FAO is commonly assessed by quantifying radiolabelled products, such as ^3^H_2_O or ^14^CO_2_, derived from the oxidation of radiolabelled FAs. Although useful, this technique is time-consuming, requiring numerous steps to prepare, purify and quantify samples. Furthermore, the use of radiolabelled material is a significant drawback, requiring special infrastructure and qualified personnel [140,141]. Alternatively, FAO can be indirectly determined via commercial colorimetric-based assays by measuring tetrazolium salt reduction linked to NADH produced during the FAO reaction. Although sometimes underperforming when quantifying FAO compared to other indirect methods, colorimetric-based assays bypass the need of using radioactive compounds [142].

State-of-the-art devices to directly quantify FAO have been introduced over the past few years and are commercially available at a number of vendors [328]. The use of these cutting-edge devices has shown several advantages, including high sensitivity and the possibility of obtaining not only real-time measurements, but also complementary mitochondrial activity read-outs in a single test run. Nevertheless, these platforms are still quite expensive and may require trained personnel.

#### 6.2.3. FA Efflux Assays

The monitoring of alterations in FA efflux of hepatocytes has been proposed as a valuable and complementary read-out to study liver steatosis in vitro [143,276,279]. Several methods have been designed to detect perturbations in lipid efflux, such as the quencher-based free FA efflux assay or the apolipoprotein B100 (APOB100) assay. The quencher-based free FA efflux assay is a highly sensitive and specific method based on the combination of a cell non-permeable quencher of extracellular free FA analogues and a fluorescent FA. The fluorescent signal obtained by this method, which is proportional to the amount of FA efflux, can be quantified using spectrophotometry or flow cytometry techniques. Alternative direct methods based on the same mechanistic principle include the use of radiolabelled FAs. Nevertheless, and despite overcoming the signal attenuation, the use of radiolabelled compounds needs to be carefully considered, since it requires the use of special infrastructure and qualified personnel [144,145].

The indirect assay APOB100 has emerged over the past years as an alternative to the direct methods due to its simplicity, sensitivity and specificity. This method is based on the quantification of APOB100, a primary structural component of other lipoproteins, such as VLDL, which plays a key role in cellular lipid efflux [329,330]. Thus, APOB100 present in the extracellular environment shows high correlation with FA efflux [143]. The levels of this protein can be determined via ELISA with commercial kits. Nonetheless, it should be stressed that the need of using antibodies increases the cost of this technique.

### 6.3. Fibrosis Assays

#### 6.3.1. Collagen Quantification Assays

##### Sirius Red Staining

The Sirius Red (SR) or Picrosirius Red (PSR) staining is a simple, low-cost, reproducible, and sensitive technique, commonly used to localise and quantify fibrillar collagen networks. This assay is based on the ability of the anionic dye SR to bind the basic amino acids located in the side chains of the collagen fibres, producing a strong red signal quantifiable via both light and fluorescence microscopy [146,147,148]. The increase in the natural birefringence of collagen bundles caused by the dye under linear polarized light allows this staining to be evaluated using linear polarized light microscopy [146,147]. Nevertheless, it is of utmost importance to note that collagen quantification under polarized light can be limited by several factors such as sample orientation, requiring specialised equipment and the need for trained personnel to guarantee the accuracy of the analysis [149].

The SR staining method offers a number of advantages, especially compared to other well- established methods, including a more stable signal when compared to Van Gieson’s trichrome staining [331], or more agility when compared to the hydroxyproline assay [150]. This, linked to the possibility of being simultaneously used with other complementary read-outs, such as Fast Green and/or immunohistochemistry [149,151], makes SR staining the backbone of integrated approaches to evaluate the liver fibrosis levels. Despite being a popular tool, certain considerations need to be kept in mind when this staining is used, including the specificity of SR to exclusively detect collagen proteins [152,153] and the capability of this dye to specifically discern between collagen types under polarised light. In this regard, while some groups associate the different colorations with a specific type of collagen fibres [332], others state that this phenomenon is a result of the collagen fibre packing density and its alignment [147,154]. A recent major breakthrough has been the development of novel assays for collagen quantification in HTP strategies, such as the commercial Sircol Collagen Assay (SCA). This colorimetric method is based on the binding properties of the SR dye, and allows for quantification of collagen in complex solutions, such as cell culture media or sample extracts [152,155].

##### Hydroxyproline Assay

The hydroxyproline assay is a method frequently used to indirectly determine the levels of total collagen. This method is based on the quantification of the hydroxyproline content, an amino acid highly abundant and almost exclusively present in the collagen molecule [333,334]. The hydroxyproline method is a multi-step process, which starts with the isolation of collagen proteins present in the sample followed by their lysis, leading to the quantification of the hydroxyproline content in the last step [156]. Several methods and techniques have been adapted for this purpose with chromatographic techniques, such as HPLC and/or LC-MS, as the gold standard procedures [335,336]. Although sensitive and valuable, cheaper and simpler alternatives based on colorimetric methods have been proposed [150,156]. Of those is an assay that determines hydroxyproline content following reaction with p-dimethylaminobenzaldehyde, which results in the formation of a coloured product measurable via spectrophotometric techniques [333,334].

Despite the high sensitivity of the hydroxyproline assay, especially when compared to other techniques, such as the SR staining [153], quantification of hydroxyproline is not without limitations. In this regard, presence of other hydroxyproline-rich proteins, such as elastine [157], can cause significant deviations of the actual collagen levels present in the sample. Linked to this lack of specificity, the hydroxyproline assay cannot distinguish between different types of collagen fibres or determine their subcellular localisation. For this reason, it is recommended to combine this assay with other methodologies, such as the SR staining. The multi-step nature of the assay and the use of toxic and relatively expensive chemicals (perchloric acid) [150,156] may also be considered as disadvantages.

##### Quantification via Immunoassays

The use of antigen recognition techniques or immunoassays is one of the major breakthroughs in the evaluation of liver fibrosis in vitro [337,338,339]. These assays are based on localising and quantifying a particular collagen type of interest by means of antibodies-based procedures, such as plate-based ELISA or immunostainings [158,159,160]. The use of immunoassays to quantify particular collagen types is a valuable and accurate methodology, especially in comparison with other well-established techniques, such as the SR staining or the hydroxyproline assay [158,159,160]. Nevertheless, its application is not exempt of caveats, including a high cost and may not allow for quantification of minor collagen types for many species. Linked to this, the high degree of homology between different collagen types still impedes the development and production of specific antibodies lacking cross-reactivity [161]. Immunoassays are not suitable for simultaneous quantification of different collagen types [162]. It is recommendable to perform additional procedures to confirm intracellular levels of these proteins [163].

#### 6.3.2. MMP and TIMP Quantification: Zymography

Zymography is a technique extensively used for evaluating ECM degradation by means of MMP activities. This technique, which represents a variation on acrylamide gel electrophoresis, is a functional and simple method based on the biological activity of the MMPs to degrade natural substrates [164,340,341]. In this method, protein separation occurs in polyacrylamide gel in the presence of a specific protease substrate. The substrate is incorporated in the gel and is degraded by proteases. After staining with Coomassie Blue, protein activity is observed by the absence of protein staining in the region where the substrate has been digested [164,341]. Variations of this technique have been developed depending on the MMP targeted, namely gelatin zymography, which uses gelatin as substrate and that is a common method for detecting gelatinases, such as MMP-2 and MMP-9 [342]; casein zymography, which is more suitable for detecting MMP-3, 10 and 7, and collagen zymography, which is more adequate to detect MMP-1 and MMP-13. Zymography is a low-cost method with high sensitivity. Gelatin zymography has the highest sensitivity and allows simultaneous determination of both active and latent forms of gelatinases. Limitations include the low number of samples processed at once and difficulties to discriminate between different classes of MMPs [164,165].

Reverse zymography is a complementary application to gelatin zymography. Reverse zymography enables the detection of endogenous TIMP, which also play a key role in the ECM homeostasis [187]. Conditioned cell culture medium is used as a source of MMPs to detect inhibitory activity. This technique has the disadvantage of displaying variable sensitivity [165,166]. Real-time zymography and real-time reverse zymography offer monitoring of the enzymatic reaction after electrophoresis with higher sensitivity [167]. Fluorescein-isothiocyanate-labelled substrates are used and the reaction is visualised using a transilluminator [164,165].

Immunoassays, such as immunoblotting and ELISA, are also capable of detecting MMPs and TIMPs with high sensitivity. However, these methods are expensive and time-consuming [165,340].

#### 6.3.3. HSC Activation Assays

##### Contraction Assay

Contraction of HSCs has been widely used as a marker of HSC activation, which is manifested during liver fibrosis [187]. Several experimental models have been focused on evaluating this process [343]. One model evaluates the reduction in the surface area of HSCs cultured on a glass coverslip in response to various agonists and inhibitors using light microscopy. However, the correlation between these quantitative measurements and the contractile force generated by HSCs has not been completely established [344,345]. HSC cultured in a monolayer on silicon-rubber is another approach for assessing HSC contraction. This model determines the wrinkling of silicon-rubber substrate using light microscopy, and it attempts to correlate the changes in HSC contractile force generation to substrate wrinkling [168,169,170]. HSC contraction has also been evaluated using a model in which HSCs in culture were grown on the top of or within gel lattices composed of collagen type I. Measurement of cellular contraction of HSCs cultured in collagen type I gel evaluates changes in gel diameter, which does not include relaxation forces and acute changes in contractile force generation. This model provides a more precise quantification of contraction and relaxation forces within the same sample compared to glass coverslip and silicon-rubber substrates [171,172,173,174]. The main concern in all models is the uncertain correlation between HSC contractile force in vitro and in vivo. Considering the advantage of closely resembling the organ of origin, primary HSC cultures have been recognised to have greater clinical relevance [343].

Functional changes associated with HSC activation can be monitored by assessing various parameters, such as cell viability, proliferation, migration and contractile response after exposure to chemicals. Thereby, HSC contraction assay is commonly combined with wound-healing assay, which evaluates the migration capacity of activated HSCs [346]. The wound healing assay consists of evaluating the HSC response to a desired compound after creating wounds by scrapping the cell monolayer using sterile microtip. Migration and contraction of activated HSCs contribute to the development of liver fibrosis.

##### α-SMA Quantification

Another marker commonly used to evaluate the early activation of HSCs is the cytoskeleton component α-SMA [187]. The assessment of α-SMA is typically performed via immunoassays by using specific antibodies [347,348,349,350]. The limitations of this method include the lack of appropriate positive and negative controls as well as standardised protocols and interpretation of results, which can lead to subjective and inaccurate conclusions [175].

## 7. Critical Parameters for Practical In Vitro Liver Toxicity Testing

**Figure 4:**
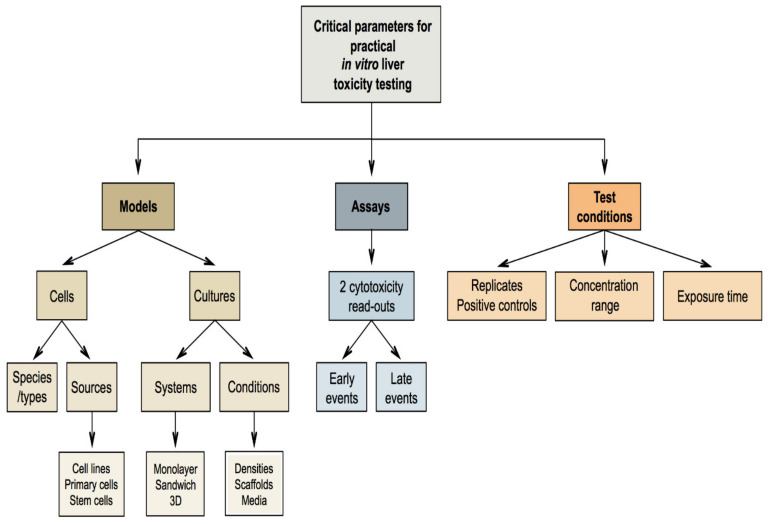
Diagram summarizing critical parameters for in vitro liver toxicity testing.

When no information on potential liver toxicity of a chemical is available, a pragmatic tiered in vitro testing strategy can be set up, in which the first tier is focused on assessing general cytotoxicity, while the second tier is aimed at identifying liver-specific toxicity. A number of practicalities need to be carefully considered when establishing such in vitro testing schemes, in particular the selection of the in vitro models, the selection of the in vitro assays and the selection of the test conditions (Figure 4).

### 7.1. Selection of the In Vitro Models

In order to be mechanistically sound and relevant, the cellular in vitro system for tier 1 testing should allow to pick up all critical events of general cytotoxicity at an in vivo-like level. Although rodent-based in vitro models are still widely being used, human-based in vitro models are obviously strongly preferred. In this respect, even for generic processes, such as inflammation, the underlying mechanisms often show poor correlation between mouse and human [7]. The cellular origin of the in vitro model is another critical parameter. Tier 1 and tier 2 testing should be ideally done using 1 single liver-based in vitro model. However, for practical reasons or specific investigations, tier 1 testing can make use of a different cell type, such as fibroblasts. After all, cytotoxicity is not a cell type-specific process, yet some tissues may be more susceptible to this process compared to others.

In recent years, so-called microphysiological systems, consisting of interacting organs-on-a-chip or tissue-engineered 3D organ constructs that use human cells, have been introduced. Such state-of-the-art in vitro systems hold great promise, especially for disease modelling [351,352,353]. However, it is not always necessary to use such complex in vitro systems for toxicity testing. In this respect, most types of liver toxicity can be more easily and reliably studied in rather simple in vitro models [353,354]. Cell lines are among the most, if not the most, frequently addressed in vitro systems in toxicology. They offer a number of advantages, especially compared to primary cells, including providing an unlimited cell supply, high reproducibility of test results and ease of use. Several human-based cell lines have repeatedly shown their value for general cytotoxicity testing, such as human embryonic kidney (HEK293) cells and T-cell leukemia Jurkat cells [353,354,355]. The human HepG2 and HepaRG liver cell lines are popular tools in in vitro liver-toxicity testing [126,356,357]. It should, however, be kept in mind that quite a few cell lines originate from cancers, implying that they may show aberrant functionality. This specifically holds true for human hepatoma HepG2 cells, which display poor biotransformation capacity, thus impeding the bioactivation of chemicals [354,358]. Cancer cells also often present altered cell death activity [359]. In fact, the background level of cell death in the selected in vitro model should be reduced as much as possible, as this may interfere with the test outcome. This is the case for conventional monolayer cultures of primary hepatocytes, which cope with significant spontaneous apoptosis and necrosis [360]. This onset of cell death is part of the general progressive loss of the in vivo-like hepatocyte phenotype at the functional and morphological level, a process called dedifferentiation. Nevertheless, primary hepatocytes and their cultures are still considered as the gold standard in vitro model for liver-specific toxicity testing [351]. A number of cell culture configurations have been implemented to counteract hepatocyte dedifferentiation, thereby enabling long-term in vivo-relevant cultivation. Such in vitro models include, but are not limited to, spheroid cultures as well as sandwich cultures of primary hepatocytes [361,362,363]. They have been shown most appropriate for testing liver-specific toxicity [118,339,364,365]. Stem cell-based in vitro models have emerged over the past 2 decades [366,367]. However, such systems, in particular induced pluripotent stem cell-based in vitro models, frequently underperform in detecting liver-specific toxicity compared to liver-derived in vitro systems [368].

For practical reasons, it is recommended to seed cells on small format culture plates, such as 96-well plates. This has a number of advantages, including reducing amounts of test material and increasing HTP potential. A critical factor is the plating density, as both too high or low densities can induce cell demise [369]. Scaffolds can also affect cell survival, with cells attached to a substratum having a higher chance to survive [370]. Furthermore, the composition of the cell culture medium is of utmost importance. A variety of culture media is commercially available, including William’s medium E, Leibovitz’s L15 medium and Dulbecco’s modified Eagle’s medium, all that are typically supplemented with a number of additives [371]. Among those, several ones counteract spontaneous cell death, such as serum [372] and glucocorticosteroids [373].

### 7.2. Selection of the In Vitro Assays

In order to sufficiently cover the mechanistic spectrum of the cytotoxicity process, at least 2 assays should be selected when testing general cytotoxicity potential of a chemical [357,374]. A commonly used combination of assays in this regard includes the MTT and LDH leakage in vitro methods. [10,195]. When using the LDH leakage assay, it is highly recommended to not use absolute testing results, as these may considerably differ between testing kits obtained from different vendors. Instead, the LDH index should be used. A typical cut-off is set at 20%, with an LDH index above this value indicating cytotoxicity [375]. MTT testing usually assesses IC_10_ and IC_50_ values, thus concentrations of the chemical that trigger cell death in 10% and 50% of the cultured cells, respectively [28,356].

### 7.3. Selection of the Test Conditions

At least 2 test runs, each including 3 biological (different cell batches) and 3 technical (different wells on a multiwell plate) repeats should be performed when no information is available regarding the cytotoxic concentration of a test chemical. A minimum of 10 concentrations spread over a vast concentration range (1 nM to 10 mM) should be tested in the first run [351,357,374]. This is narrowed down, usually within the µM range, in the second run and may be optimised in additional test runs. An even more important parameter is exposure time [374]. Alterations in gene expression patterns can be induced and detected as early as 1 h after exposure of cells to test chemicals and barely vary with increasing concentration for some toxicological effects [376]. In most general cytotoxicity test procedures, however, exposure times up to 72 h are applied [357,374]. As many cytotoxicity tests are based on assessing release of substances from cells into the cell culture medium as a function of time, the frequency of cell culture media renewal and, linked to this, the time of sampling should be carefully selected. In this regard, while some protocols foresee cell culture medium renewal every 2 or 3 days, others, in particular those using primary cells, require a change of cell culture medium every day. Furthermore, kinetic aspects should not be ignored. The actual concentration inducing the adverse effect is not merely determined by the quantity of the chemical added to the cell culture medium. Processes, such as binding to plastic cell culture plates or cell culture medium constituents as well as evaporation, can cause significant deviations in the actual cellular exposure concentrations [377,378,379].

Appropriate controls are indispensable for correct interpretation of in vitro test results. Many chemicals are not, or only partly, soluble in cell culture media and require a co-solvent, such as ethanol or DMSO. Ethanol is a known cytotoxicant, but DMSO is often added to cell culture media because of its beneficial effects on cellular functionality [353]. Nevertheless, DMSO may also cause cell damage [380]. It is therefore strictly necessary to include a solvent control when using organic solvents for test chemicals. Concomitantly, chemicals well-known to induce the toxicity biomarker concerned should be included. Typical positive controls for MTT and LDH leakage testing are tamoxifen [28] and sodium lauryl sulphate [375], respectively. Cyclosporine A, valproic acid and methotrexate can be used to induce potent cholestatic, steatotic and fibrotic responses in vitro, respectively [143,278,381,382]. An obvious negative control is the cell culture medium, yet it may be advisable to address specific chemicals as true negative controls, such as mannitol for general cytotoxicity testing [195]. Structural congeners with well-delineated toxicity profiles are convenient negative controls for testing liver-specific toxicity. A typical example includes structural congeners of the cholestatic drug bosentan, namely sitaxentan and ambrisentan, which are hepatotoxic through a non-cholestatic mechanism and non-hepatotoxic, respectively [383].

## 8. Conclusions

Predictive toxicology based upon mechanistic information has become a critical aspect of chemical risk assessment in the last two decades. A major step in this direction came with the introduction of adverse outcome pathways (AOPs). An AOP refers to a conceptual construct that portrays existing knowledge concerning the linkage between a direct molecular initiating event and an adverse outcome at a biological level of organisation relevant to risk assessment. In practice, an AOP is a graphical scheme that represents the mechanisms driving a specific type of adverse effect. Each AOP consists of a series of key events that are connected through key event relationships. A key event represents a measurable change in a biological state that is essential, but not necessarily sufficient, for progression to the adverse outcome. AOPs have become major tools in the fields of toxicology and risk assessment [384,385,386,387,388]. AOPs have been proposed for a multitude of toxicological endpoints, including general cytotoxicity, cholestasis, liver steatosis and liver fibrosis [143,381,382,389]. Among the many applications, AOPs can be used as the conceptual basis for the development of new in vitro tests or testing approaches that detect specific key events. The advent of such assays and testing schemes that are mechanistically anchored in AOPs goes hand in hand with the emerging tendency to step away from 1-to-1 replacement of animal studies for toxicity testing with single non-animal methods [390]. Instead, animal studies for toxicity testing should be replaced by batteries of non-animal assays encompassing the full mechanistic spectrum as depicted in AOPs and thus reflecting the in vivo complexity of adversity [195,385,388]. As matter of fact, this is the rationale for the 2-tiered testing approach proposed in the current paper [195,357,374]. Tier 1 testing should include assays that detect at least 2 key events of general cytotoxicity, typically an MTT assay (mitochondrial dysfunction) and an LDH leakage assay (cell plasma membrane damage). Tier 2 testing should be ideally built on a series of assays each that monitors a key event in the respective AOP [143,381,382]. This can be accomplished with a number of cutting-edge devices introduced over the past few years and commercially available at a number of vendors. Such devices allow to study multiple read-outs and, thus, key events simultaneously in real time in a single test run typically in HTP strategies. Although extremely valuable, these devices are still quite expensive and may not cover the more specific key events. In this respect, it may not be necessary to detect all key events in in vitro liver toxicity testing [391]. For feasibility reasons, focus can indeed be put on rate-limiting key events, which can be identified by kinetic modelling, including the establishment of concentration–response relationships. Furthermore, it should be stressed that in vitro assays are just 1 type of non-animal testing tools. Other methodologies to detect (rate-limiting) key events, such as in silico (computational testing) and in chemico (abiotic chemical reactivity testing) techniques, are also routine constituents of advanced batteries for testing chemicals [392]. Besides being pragmatic, relevant and integrative, such test batteries are highly agile and versatile in that they are typically applicable to any type of chemical and can be readily adapted to the needs of specific chemical sectors. These test batteries should be combined with microarray or ribonucleic acid (RNA) sequencing technology, which allows to create transcriptomic signatures for specific types of toxicity [393,394,395,396]. Such transcriptomic blueprints are available for the different liver toxicity AOPs, albeit not always with high predictivity, especially in the case of cholestasis [382,394]. Predictivity could be increased by follow-up with an AOP-based test battery. This not only allows (qualitative) hazard identification, but equally enables (quantitative) hazard characterisation, which in turn may support potency ranking of chemicals. This can form the backbone of integrated approaches to testing and assessment (IATA), which are already available for a number of toxicity endpoints, but not yet for liver toxicity. The outcome of tiered and AOP-based testing should be subjected to weight-of-evidence analysis and needs to be accompanied by tailored exposure assessment for proper risk characterisation [386,393]. Recently, artificial intelligence has entered the liver toxicity and risk assessment arena. Although still in its infancy, expectations are high and major breakthroughs are anticipated in the upcoming years. Through machine and deep learning, potential liver toxicity induced by chemicals can be predicted with high accuracy and predictivity [397,398]. This will also allow to identify knowledge and data gaps, which can be filled by targeted testing using customized test batteries, such as the two-tiered approach presented in this paper. Research in this direction should be strongly encouraged, as it helps to meet the ever-increasing safety requirements for chemicals, while reducing and replacing the use of laboratory animals.

## Figures and Tables

**Table 1:** Advantages and limitations of in vitro assays to study general cytotoxicity.

	Assay	Advantages	Limitations	References
Membrane integrity	LDH leakage assay	Sensitivity, agility and low cost. Multiple time points analysis in a single test run. High stability of the LDH enzyme.	Interference of cell culture components/test chemical with the LDH stability.	[9,10,11,12,13]
	Calcein-AM assay	Simplicity, safety and low cost. Suitability for HTP strategies. Possibility of combining with other read-outs in a single test run.	Spontaneous leakage of the dye. Stability of the signal. Limited dye uptake in certain cell types. Potential signal overlap between the calcein and the test chemical.	[14,15,16]
	Protease activity assay	Multiple time points analysis in a single test run. Possibility of combining with other read-outs in a single test run. Suitability for HTP strategies.	Interference of cell culture components with the protease activity.	[17,18]
	Trypan blue exclusion assay	Agility, simplicity and low cost.	Intra-operator/inter-operator variability. Dichotomic nature of the results. Sensitivity can be compromised by the concentrations and exposure time to the dye.	[19,20,21]
Mitochondrial functionality	Tetrazolium salt assays	Simplicity and reproducibility. Low cost.	Variable results depending on the cell culture stage/cell type. Cell culture components/test chemical can catalyse MTT reduction. Potential cytotoxicity of the reagents. Lytic endpoint methodology.	[9,22,23,24,25,26]
	Resazurin reduction assay	Agility, sensitivity and simplicity. Possibility of combining with other read-outs in a single test run. Suitability for HTP strategies. Multiple time points analysis in a single test run.	Potential cytotoxicity of the reagents. Optimisation for each cell type. Interference of cell culture components with the colorimetric signal.	[14,27,28,29,30,31,32]
	ATP content assay	Agility, sensitivity and reproducibility. Stability of the signal. Low background noise. Detection of early cytotoxicity. Applicable to evaluate 3D cultures.	Test chemical and/or cell culture conditions can alter luciferase activity. Lytic endpoint methodology. Levels of ATP can be compromise by ATPases present in the media. Expensive.	[10,27,33,34,35,36,37,38]
	Mitochondrial membrane potential evaluation: fluorescent probe-based assays	Reliable indicator of mitochondrial functionality. Multiplatform evaluation (flow cytometer, fluorescence microscope or plate reader).	Low sensitivity and non-specificity of certain probes. Potential cytotoxicity of the probes. Requires the use of pharmacological controls and/or complementary probes.	[39,40,41,42,43]
Oxidative stress	DCFH_2_-DA fluorescence probe-based assay	Agility. Multiplatform evaluation (flow cytometer, fluorescence microscope or plate reader).	Low sensitivity and non-specificity of certain probes. Artificial amplification of the signal. Spontaneous leakage of certain probes.	[44,45,46,47,48,49,50,51,52]
	DHE/Mito-HE fluorescence probe-based assays	Agility. Multiplatform evaluation (flow cytometer, fluorescence microscope or plate reader). Evaluation of mitochondrial ROS levels.	Low sensitivity and non-specificity of the probe. Optimisation of the probe concentration.	[53,54,55,56,57]
	Lipid peroxidation: MDA/TBARS assay	Agility, simplicity, low cost.	Low specificity, artificial amplification of the signal. Relatively low detection limit.	[58,59,60,61,62]
	Enzymatic antioxidants activity assays	Specificity. Quantitative and functional nature of the results.	Lytic endpoint methodology. No information about cellular localisation. Requires the use of control conditions for isoform signatures.	[63,64,65]
Cell death	Annexin V staining assay	Sensitivity. Multiplatform evaluation (flow cytometer, fluorescence microscope). Possibility of combining with other read-outs in a single test run. Application in tissues and whole-body level.	Background signal. Requirement for calcium ions in mM concentrations.	[66,67,68,69,70,71,72]
	PI dye uptake assay	Versatility and low cost. Multiplatform evaluation (flow cytometer, fluorescence microscope). Possibility of combining with other read-outs in a single test run. Suitability for HTP strategies.	Optimisation of the dye concentration, incubation time and washing steps. Potentially mutagenic effect of the dye.	[69,73,74]
	Caspase activity assays	Agility, reproducibility and sensitivity. Multiplatform evaluation (flow and laser scanning cytometer). Possibility of combining with other read-outs in a single test run.	Non-specific for a particular caspase. Lytic endpoint methodology.	[75,76,77,78,79]
	TUNEL assay	Agility, sensitivity and simplicity. Detection of early stages of apoptosis. Multiplatform evaluation (light and fluorescence microscope, flow cytometer). Possibility of combining with other read-outs in a single test run.	Expensive. Time-consuming. Subjected to false positive results.	[68,80,81,82,83,84]
Miscellaneous	Neutral red uptake assay	Versatility, sensitivity and low cost. Stability of the signal. Suitability for HTP strategies.	Optimisation of the dye concentration and incubation time. Impact of the test chemical on the dye activity. Lytic endpoint methodology.	[85,86,87,88,89,90,91,92,93,94,95]

ATP: adenosine triphosphate; Calcein-AM: calcein-acetoxymethyl; DCFH_2_-DA: 2′,7′-dichlorodihydrofluorescein diacetate; DHE: dihydroethidium; HTP: high-throughput; LDH: lactate dehydrogenase; MDA: malondialdehyde; MTT: 5-(3-carboxymethoxyphenyl)-2-(4,5-dimethylthiazoly)-3-(4-sulfophenyl)-tetrazolium; PI: propidium iodide; ROS: reactive oxygen species; TBARS: thiobarbituric acid reactive substance; TUNEL: terminal deoxynUcleotidyl transferase dUTP Nick End Labeling.

**Table 2:** Advantages and limitations of in vitro assays to study liver-specific toxicity.

	Assay	Advantages	Limitations	References
Cholestasis	Transporter inhibition assays	Tauro-nor-THCA-24-DBD: sensitivity for BSEP inhibition assessment. CLF: agility and low cost. CDFDA: robustness. TCA: sensitivity for NTCP uptake activity. Estradiol-17β-glucuronide and CCK8: selectivity of the probe substrates for OATP1B1 and OATP1B3 transporters.	Tauro-nor-THCA-24-DBD: uptake of substrate by NCTP must be considered for interpretation of results. CLF: prone to artefacts and test compound interference. CDFDA: test compound interference is possible. TCA: safety concerns when using radiolabelled-TCA. Estradiol-17β-glucuronide and CCK8: substrate selectivity depends on concentration.	[96,97,98,99,100,101]
	Drug-induced cholestasis assay	Early assessment and prediction of DIC risk. Determination of DICI. Mechanistic information on cholestatic compounds.	Not suitable for long-term toxicity analysis.	[102,103,104]
Steatosis	Oil Red O staining	Low cost and simplicity. Potential quantification using computed-based software. Multiplatform evaluation (light and fluorescence microscope, plate reader). Possibility of combining with other read-outs in a single test run.	Specificity. Stability of the signal. Preparation of the solutions ex tempore. Not suitable for combining with alcohol-based fixatives and paraffin-embedded procedures.	[105,106,107,108,109,110,111,112,113]
	Nile Red staining	Simplicity, reproducibility and agility. Low background. Detection of specific lipids (solvatochromic property). Application on fixed and live cells. Multiplatform evaluation (fluorescence microscope, flow cytometer and plate reader). Preparation of the dye in aqueous medium. Suitability for HTP strategies.	Specificity. Not suitable for multicolour imaging.	[114,115,116,117,118,119,120,121,122,123] [124,125]
	BODIPY 493/503 staining	Agility. Application on fixed and live cells. Multiplatform evaluation (fluorescence microscope, flow cytometer). Possibility of combining with other read-outs in a single test run. Evaluation of both live and fixed cells. Stability of the dye.	Optimisation of the filters. Background signal. Intensity and stability of the signal.	[125,126,127,128,129,130,131,132,133]
	Absolute lipid quantification assays	Specificity and sensibility. Quantitative nature of the results. Commercial kits available. Suitability for HTP strategies.	Time-consuming multi-step procedure. Use of harmful reagents. No information about cellular localisation.	[134,135,136,137,138,139]
	FA oxidation assays	Direct and indirect quantification of FAO. Multiplatform evaluation (fluorescence and radiometric devices). Commercial kits available.	Time-consuming method multi-step procedure. Sensitivity. Use of radiolabelled compounds.	[140,141,142]
	FA efflux assays	Direct and indirect quantification of FA efflux. Multiplatform evaluation (flow cytometer and spectro-radiometric devices). Commercial kits available.	Use of radiolabelled compounds. Stability of the signal (fluorescence methods). Expensive.	[143,144,145]
Fibrosis	Sirius Red staining	Agility, sensibility, reproducibility, simplicity and low cost. Multiplatform evaluation (light, fluorescence and polarized microscope). Stability of the signal. Possibility of combining with other read-outs in a single test run. Suitability for HTP strategies.	Specificity. Special equipment and qualified personnel.	[146,147,148,149,150,151,152,153,154,155]
	Hydroxyproline assay	Sensitivity.	Time-consuming method multi-step procedure. Specificity. Lytic endpoint methodology. No information about cellular localisation. Use of toxic and expensive reagents.	[150,153,156,157]
	Collagen quantification via immunoassays	Sensitivity and specificity. Discrimination of specific collagen types.	Expensive. Potential cross-reactivity of the antibodies. Lack of antibodies against minor collagen types. Not allows simultaneous detection of several collagen types.	[158,159,160,161,162,163]
	MMP and TIMP quantification: zymography	Detection of specific MMP and TIMP. Low cost. Potential application in real-time procedures.	Zymography: time-consuming multi-step procedure. Reverse zymography: sensitivity.	[164,165,166,167]
	HSC activation assays	Contraction assay: functional assay. α-SMA quantification: reliable marker of activated HSC.	Contraction assay: uncertain correlation between HSC contractile force in culture and in vivo. α-SMA quantification: lack of standardised interpretation.	[168,169,170,171,172,173,174,175]

BSEP: bile salt export pump; BODIPY 493/503: 4,4-difluoro-1,3,5,7,8-pentamethyl-4-bora-3a,4a-diaza-s-indacene 495/503; CCK8: cholecystokinin-octapeptide; CDFDA: 5(6)-carboxy-2′,7′-dichlorofluorescein diacetate; CLF: cholyl-lysyl-fluorescein; DIC: drug-induced cholestasis; DICI: drug-induced cholestatic index; FA: fatty acid; FAO: fatty acid oxidation; HSC: hepatic stellate cell; HTP: high-throughput; MMPs: matrix metalloproteinases; NTCP: sodium taurocholate co-transporting polypeptide; OATP: organic anion transporting polypeptide; Tauro-nor-THCA-24-DBD: tauro-nor-*N*-(24-[7-(4-*N*,*N*-dimethylaminosulfonyl-2,1,3-benzoxadiazole)]-amino-3α,7α,12α-trihydroxy-27-nor-5β-cholestan-26-oyl)-2′-aminoethanesulfonate; TCA: taurocholate; TIMPs: tissue inhibitor of metalloproteinases; α-SMA: alpha-smooth muscle actin.
